# Contrastive learning and subtyping of post-COVID-19 lung computed tomography images

**DOI:** 10.3389/fphys.2022.999263

**Published:** 2022-10-11

**Authors:** Frank Li, Xuan Zhang, Alejandro P. Comellas, Eric A. Hoffman, Tianbao Yang, Ching-Long Lin

**Affiliations:** ^1^ Roy J. Carver Department of Biomedical Engineering, University of Iowa, Iowa City, IA, United States; ^2^ IIHR-Hydroscience and Engineering, University of Iowa, Iowa City, IA, United States; ^3^ Department of Mechanical Engineering, University of Iowa, Iowa City, IA, United States; ^4^ Department of Internal Medicine, University of Iowa, Iowa City, IA, United States; ^5^ Department of Radiology, University of Iowa, Iowa City, IA, United States; ^6^ Department of Computer Science, University of Iowa, Iowa City, IA, United States

**Keywords:** computed tomography, post-COVID-19, contrastive learning, cluster analysis, small airways disease, long Covid, PASC

## Abstract

Patients who recovered from the novel coronavirus disease 2019 (COVID-19) may experience a range of long-term symptoms. Since the lung is the most common site of the infection, pulmonary sequelae may present persistently in COVID-19 survivors. To better understand the symptoms associated with impaired lung function in patients with post-COVID-19, we aimed to build a deep learning model which conducts two tasks: to differentiate post-COVID-19 from healthy subjects and to identify post-COVID-19 subtypes, based on the latent representations of lung computed tomography (CT) scans. CT scans of 140 post-COVID-19 subjects and 105 healthy controls were analyzed. A novel contrastive learning model was developed by introducing a lung volume transform to learn latent features of disease phenotypes from CT scans at inspiration and expiration of the same subjects. The model achieved 90% accuracy for the differentiation of the post-COVID-19 subjects from the healthy controls. Two clusters (C1 and C2) with distinct characteristics were identified among the post-COVID-19 subjects. C1 exhibited increased air-trapping caused by small airways disease (4.10%, *p* = 0.008) and diffusing capacity for carbon monoxide %predicted (DLCO %predicted, 101.95%, *p* < 0.001), while C2 had decreased lung volume (4.40L, *p* < 0.001) and increased ground glass opacity (GGO%, 15.85%, *p* < 0.001). The contrastive learning model is able to capture the latent features of two post-COVID-19 subtypes characterized by air-trapping due to small airways disease and airway-associated interstitial fibrotic-like patterns, respectively. The discovery of post-COVID-19 subtypes suggests the need for different managements and treatments of long-term sequelae of patients with post-COVID-19.

## Introduction

As of September 2022 over ninety million cases of coronavirus disease 2019 (COVID-19) in the United States have been reported to the Centers for Disease Control and Prevention (CDC) (Centers for Disease Control and Prevention, 2022). It has been shown from a meta-analysis that the patients who recovered from COVID-19 experienced several long-term physical, cognitive, and mental health symptoms (Han et al., 2022), given the diagnosis long COVID, post-acute COVID-19 syndrome (PACS), or post-acute sequelae of COVID-19 (PASC) (Nalbandian et al., 2021; Proal and VanElzakker, 2021; Sugiyama et al., 2022; Tran et al., 2022). Since the lung is the most common site of infection of the severe acute respiratory syndrome coronavirus 2 (SARS-CoV-2), impaired lung function due to pulmonary fibrosis and airway injury is frequently observed in patients with post-COVID-19 (Wang et al., 2020; Cho et al., 2022; Jia et al., 2022). Thus, pulmonary sequelae may manifest persistently in COVID-19 survivors.

Chest X-ray and computed tomography (CT) scans are widely used to examine patients with COVID-19 (Mukherjee et al., 2021; Song et al., 2021; Wang et al., 2021; Zou et al., 2021; Mahbub et al., 2022; Santosh et al., 2022). With medical care and management of post-COVID-19 subjects being recognized as a top research priority by professional societies, follow-up evaluations of COVID-19 survivors based on chest X-ray or CT scans along with clinical assessment have been recommended (Zheng et al., 2020). We hypothesize that post-COVID-19 subtypes exist and can be differentiated by contrastive self-supervised learning of 2D lung images.

Contrastive learning has recently gained attention in the computer vision community because of its success in self-supervised representation learning. Contrastive learning is considered as learning by comparing the similarities between image pairs and the image pairs are generated by data augmentation techniques. That is, by contrasting between images of positive and negative pairs, representations of positive pairs will be attracted together while representations of negative pairs will be repelled far apart (Le-Khac et al., 2020). Chen et al. proposed a simple framework for contrastive learning of visual representations (SimCLR) to learn representations of the images and use them for downstream tasks, e.g. predictive tasks and clustering, to achieve state-of-the-art results (Chen et al., 2020; Li et al., 2021).

CT images acquired at inspiration and expiration reveal different lung disease phenotypes such as emphysema and air trapping, respectively. Registration of inspiratory and expiratory CT images can further identify the extent of functional small airways disease (Galbán et al., 2012). In this study, we introduced a new lung volume transform to the data augmentation techniques in the SimCLR model to learn from positive pairs of CT images at inspiration and expiration, so that the extracted representations not only capture disease phenotypes at various lung volumes but also are invariant to the lung volume of input image - a volume-independent feature. Moreover, 3D CT images were used to construct composite 2D images, mimicking chest X-rays, as inputs to the model, in hope that the model might be applicable to chest X-rays *via* transfer learning in the future.

The objective of this study is to construct a contrastive learning model that can differentiate post-COVID-19 subjects from healthy (no SARS-CoV-2 infected) subjects and to identify post-COVID-19 subtypes using lung CT scans. The discovery of post-COVID-19 subtypes may assist with the management and treatment of long-term sequelae of post-COVID-19 subjects.

## Meterial and methods

### Human subject data and image processing

In this study, a total of 245 de-identified subjects were selected for analysis. Among those, 140 subjects, who were tested positive for SARS-CoV-2 between June and December 2020, visited a post-COVID-19 outpatient clinic at University of Iowa hospitals and clinics for follow up. The mean time interval between the diagnosis of COVID-19 and the first visit to the post-COVID-19 clinic was 112.99 days. The other 105 subjects were healthy controls who were not infected with SARS-CoV-2. We retrospectively collected inspiratory and expiratory quantitative CT image data acquired at breathing stages of total lung capacity (TLC) and residual volume (RV), demographic data, and pulmonary function test (PFT) results. The demographic data and PFT measures for each stratum are shown in Table 1. The study was approved by Institutional Review Board at the University of Iowa and written informed consents were obtained from all the patients included in the study. 205 of the 245 subjects have been previously reported, and asthma was the most common coexisting pulmonary disorder (26%) among the subjects with PASC (Cho et al., 2022). This prior article analyzed the traditional clinical and imaging metrics of the subjects, whereas in this study we developed a contrastive learning model to detect imaging features and then performed cluster analysis.

**TABLE 1:** Demographic and PFT data for all the subjects.

	Post-COVID (*n* = 140)		Control (*n* = 105)			All (*n* = 245)	
	Mean	SD	Mean	SD	p	Mean	SD
**Age (yrs.)**	45.88	15.93	44.59	14.05	0.504	45.32	15.13
**BMI**	32.13	7.58	25.73	3.59	<0.001	29.74	8.38
**FVC %Predicted**	97.81	15.25	101.00	10.37	0.064	98.83	14.11
**FEV1 %Predicted**	97.60	14.75	99.50	10.71	0.263	97.85	14.09
**RV/TLC (%)**	29.28	8.94	28.39	7.74	0.424	28.75	9.79
**TLC (L)**	5.56	1.35	5.83	1.24	0.117	5.70	1.33
**RV (L)**	1.57	0.56	1.66	0.62	0.254	1.63	0.69
**DLCO %Predicted**	97.74	22.00	89.92	13.21	0.001	93.50	20.11
	**Percentage**		**Percentage**		**p**	**Percentage**	
**Female (%)**	0.66		0.51		0.025	59.50	

CT images were rescaled with a range from a minimum value of zero (air) to a maximum value of one (tissue) to account for the scanner difference (Choi et al., 2014; Kim et al., 2014) and then were masked by the lung masks obtained from the VIDA Vision software (VIDA Diagnostics, Coralville, Iowa). Subsequently, average was taken from the slices in the coronal plane to convert the original 3D CT images to a single 2D image, mimicking a chest X-ray image. We used SimpleITK (version 2.1) for further image processing.

### Contrastive learning model

There were 490 2D images in the dataset which contained 245 TLC images and 245 RV images. The dataset was then randomly split into a training set and a testing set, which comprised of 440 and 50 images, respectively.

**FIGURE 1:**
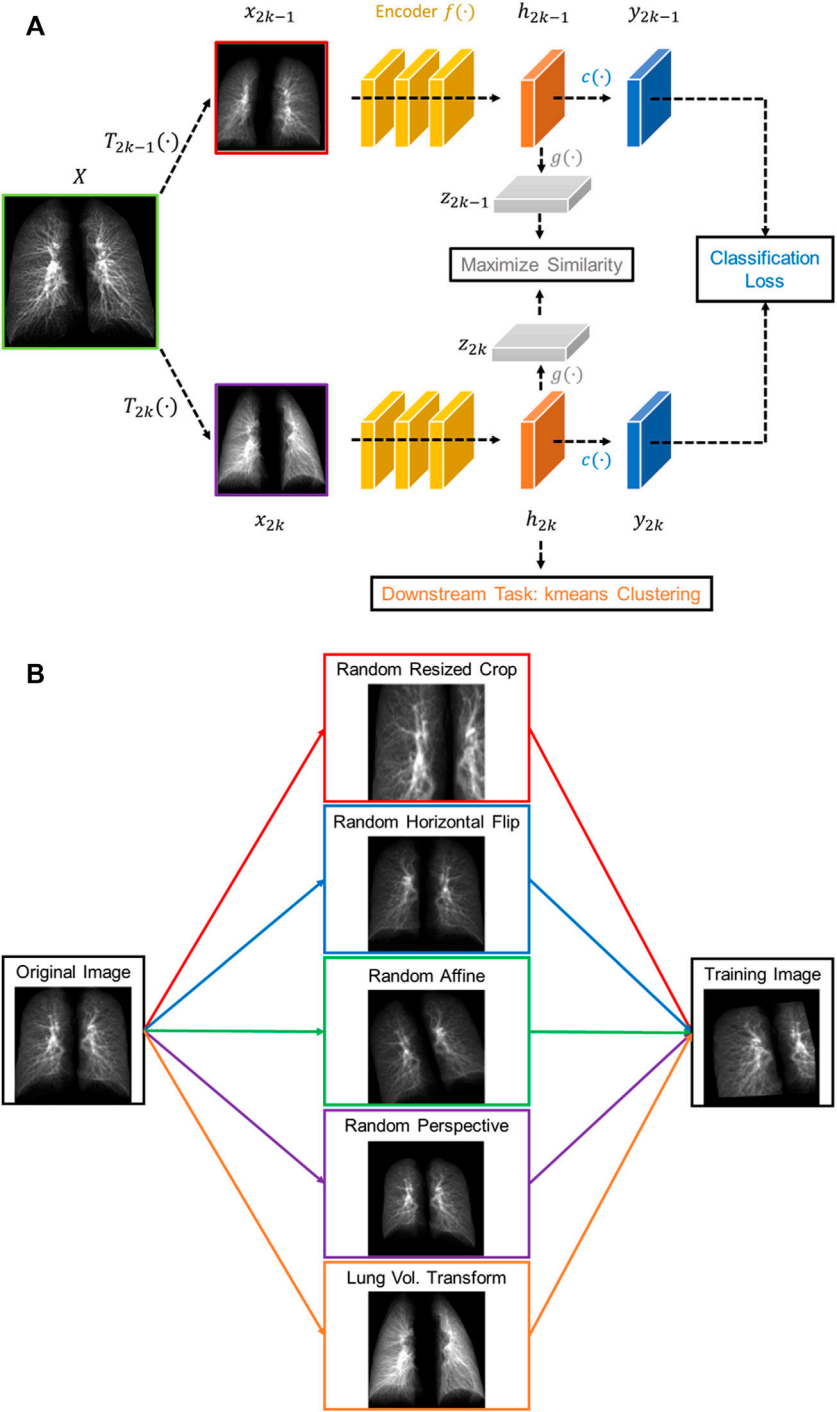
**(A)** The structure of the proposed volume-independent contrastive learning model. **(B)** Illustrations of the transforms applied to the training images.

The proposed contrastive learning model is based upon SimCLR (Chen et al., 2020), which learns representations by contrasting image pairs and is implemented with a classifier which can differentiate post-COVID-19 subjects from healthy controls. The structure of the model is presented in Figure 1A. First, the contrasting image pair is generated by two transformations, $T_{2k-1}\left(∙\right)$ and $T_{2k}\left(∙\right)$ , which are composite transforms comprised of random resized clop, random horizontal flip, random affine transform, and random perspective transform (Figure 1B). Furthermore, we applied a volume transform of which a certain probability for the input image of $T_{2k}\left(∙\right)$ is transformed to the image at its counterpart lung volume (i.e. TLC image being replaced by RV image and *vice versa*). The probability was set at 0.4 in this study. Next, the representations $h_{2k-1}$ and $h_{2k}$ are extracted by the encoder f(·) comprising the pre-trained convolutional layers of the ResNet18, which is able to achieve good performance in predicting COVID-19 subjects with a relatively small set of parameters as compared with other state-of-the-art convolutional neural networks (Pham, 2020). $h_{2k-1}$ and $h_{2k}$ are mapped to the space ( z ) where contrastive loss is applied by the projection head g(·) , which is a multilayer perceptron with one hidden layer. In addition, they are fed into a sigmoid classifier c(·) for differentiating the post-COVID-19 subjects from the healthy controls, where the history of being diagnosed with COVID-19 was treated as the ground truth for the classification.

The total loss ( $L_{total}$ ) is the weighted average of the contrastive loss ( $L_{contrastive}$ ) and classification loss ( $L_{class}$ ) as defined in Eq. 1. The similarity ( $s_{i,\;j}$ ) between $z_{i}$ and $z_{j}$ is maximized by minimizing the normalized temperature-scaled cross entropy loss (NT-Xent, Eq. 3). $L_{contrastive}$ is computed at both l(i, j) and l(j, i) in a mini-batch (Eq. 4). For more information of the contrastive loss, please refer to Chen et al. (2020). On the other hand, $L_{class}$ is the binary cross entropy loss of sample i and sample j in a mini-batch (Eq. 5).

$$L_{total}=w_{1}L_{contrastive}+w_{2}L_{class}$$ (1)

$$s_{i,\;j}=\frac{z_{i}z_{j}}{\left\|z_{i}\right\|\left\|z_{j}\right\|}$$ (2)

$$l\left(i,\;j\right)=-\log\frac{\exp\left(s_{i,\;j}/\tau \right)}{\sum_{k=1}^{2N}1_{\left[k\neq i\right]}\exp\left(s_{i,\;k}/\tau \right)}$$ (3)

$$L_{contrastive}=\frac{1}{2N}\sum_{k=1}^{N}\left[l\left(2k-1,\;2k\right)+l\left(2k,\;2k-1\right)\right]$$ (4)

$$L_{class}=-\frac{1}{2N}\left[\begin{array}{c} \sum_{k=1}^{N}y_{2k}\log\left(p\left(y_{2k}\right)\right)+\left(1-y_{2k}\right)\log\left(1-p\left(y_{2k}\right)\right)+\\ y_{2k-1}\log\left(p\left(y_{2k-1}\right)\right)+\left(1-y_{2k-1}\right)\log\left(1-p\left(y_{2k-1}\right)\right) \end{array}\right]$$ (5)

where $w_{1}$ and $w_{2}$ are weights for $L_{class}$ and $L_{contrastive}$ , respectively, and N is the sample size of a mini-batch. The model was built using Pytorch 1.11 and trained with NVIDIA GEFORCE RTX 2080 Ti graphic card. The training detail was documented in the supplementary material.

In summary, our contrastive learning model introduces two new components to SimCLR: a lung volume transform to learn latent representations of phenotypes from inspiration and expiration CT images and a cross entropy loss to differentiate the post-COVID-19 subjects from the healthy controls.

### Identification of subject-clusters

The latent representations ( h ) of TLC and RV images that belonged to the same subjects were concatenated and K-means clustering was applied to search for the subject-clusters within the concatenated latent representations. The number of subject-clusters was determined by evaluating the inter-cluster and intra-cluster variability. Moreover, the inter-cluster differences in terms of the clinical and imaging-based variables were examined. The clinical variables include sex, age, body mass index (BMI), FVC %predicted, FEV_1_ %predicted, diffusing capacity for carbon monoxide %predicted (DLCO %predicted), TLC volume, RV volume, and the ratio between RV volume and TLC volume (RV/TLC). The imaging variables include emphysema percentage with respect to the total lung volume at TLC (Emph%), air trapping percentage due to functional small airways disease with respect to the total lung volume at RV (AirT_fSAD%) (Galbán et al., 2012), tissue percentage with respect to the total lung volume at TLC (Tissue_TLC%), tissue percentage with respect to the total lung volume at RV (Tissue_RV%), ground glass opacity percentage with respect to the total lung volume at TLC (GGO%), and bronchovascular pattern percentage with respect to the total lung volume at TLC (Bronchovascular%). Emph%, AirT_fSAD%, Tissue_TLC%, and Tissue_RV% were derived using our in-house software (Haghighi et al., 2018; Haghighi et al., 2019) while GGO% and Bronchovascular% were computed using a texture analysis, called Adapted Multiple Feature Method (AMFM) (Uppaluri et al., 1999).

### Statistical analysis

Pairwise deletion was used to handle any missing data. Numbers of missing data for the variables analyzed in this study are shown in Supplementary Table S1. The differences between the means of independent groups were analyzed by Welch’s ANOVA with the Games-Howell method for post-hoc pairwise tests. Chi-square test was used to examine the relationships between two categorical variables. The data points which are three standard deviations greater or smaller than the mean are treated as outliers and are excluded from the analysis. The statistical significance level α was set at 0.05. The statistical analyses were conducted using SciPy 1.4.1 and Pingouin 0.3.4 in Python 3 packages.

## Results

The contrastive deep learning and the following statistical analyses were conducted for the 245 subjects. Among them, 140 subjects (57.14%) were the post-COVID-19 patients and 105 (42.86%) the healthy controls. The post-COVID-19 subjects had greater BMI and DLCO% predicted, and lower FVC %predicted. In addition, they had higher proportion of females, compared with the control group (Table 1).

### Performance of the contrastive learning model

An averaged accuracy of 97.74% and 90.00% was achieved on a five-fold cross-validation analysis and on the testing dataset, respectively. Moreover, an area under the receiver operating characteristic curve (AUC) of 0.98 was achieved on the testing dataset (Supplementary Figure S1).

**FIGURE 2:**
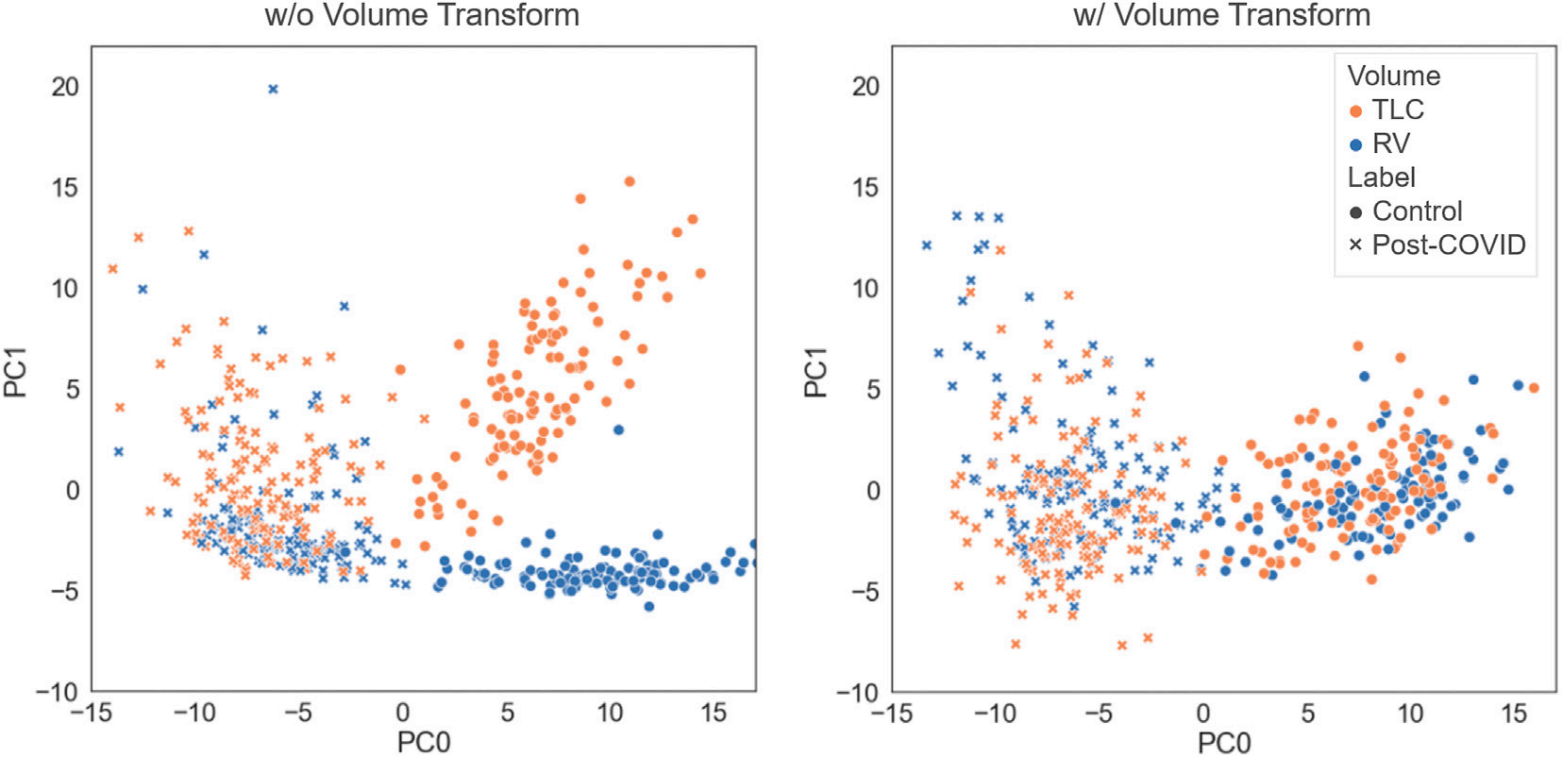
Comparison of the latent representations generated by the models trained with and without the volume transform. The data points were projected on the first and second principal axes.

We tested how performing the volume transform would affect the latent representation space. As illustrated in Figure 2, the models trained with and without the volume tranform had good performance in differentating the post-COVID-19 subjects from the control group. The model without volume transform achieved an accuracy on test data of 92% (AUC = 0.97) which is similar to the model with volume transform (AUC = 0.98). However, the latent representations of TLC and RV images generated by the model without volume transform did not share the similar features (i.e. clusters of TLC and RV images were formed) while those generated by the model with volume transform did.

### Characteristics of subject-clusters

**FIGURE 3:**
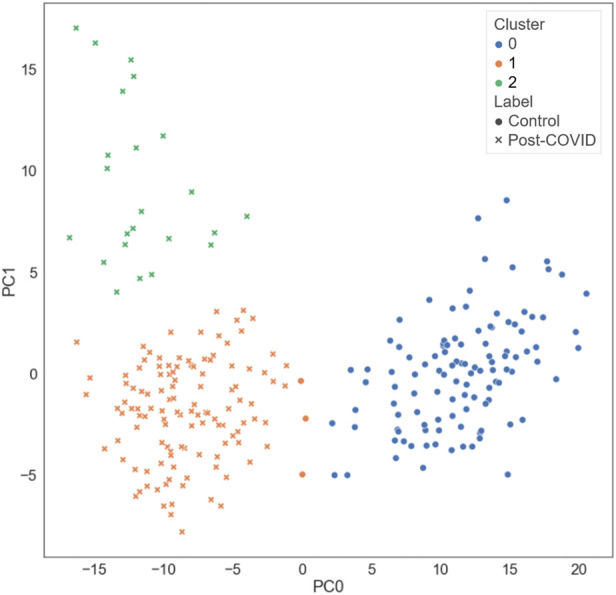
The distribution of the different types of subjects projected on the first and the second principal axes.

**FIGURE 4:**
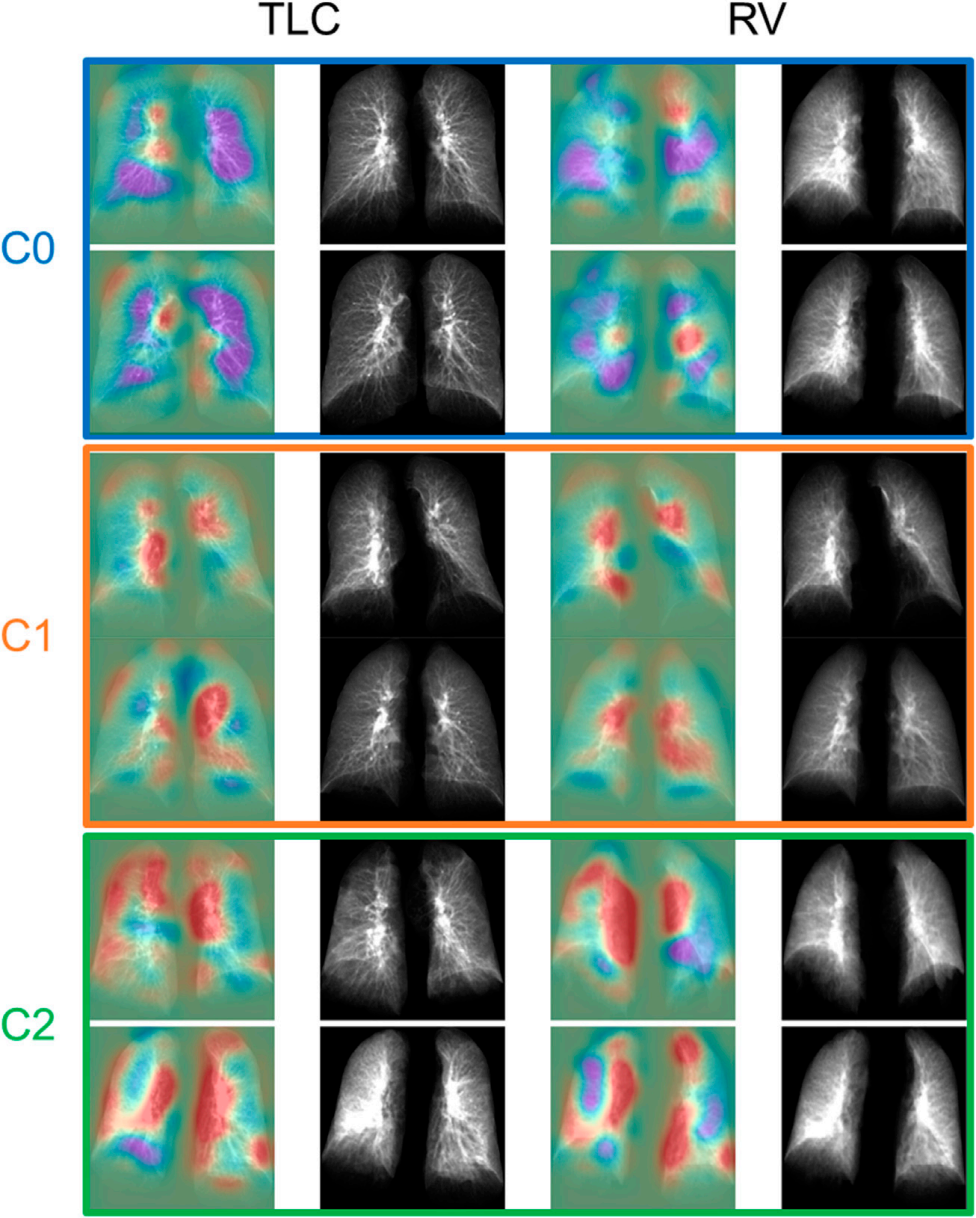
The TLC and RV images of the representative subjects for each cluster. The first and the third column showed the activation maps which indicated the important regions for determining if the subjects were post-COVID-19 subjects (red) or control subjects (purple).

Three clusters (C0-C2) were identified by k-means clustering in the feature space of latent representations of the images ( h ). There were 102, 120, and 23 subjects in C0, C1, and C2, respectively. Based on our sample size of 245 and 3 clusters, the sample size is sufficient to detect medium effect size with power of 0.95 and type one error rate of 0.05. The distribution of the different types of subjects projected in a 2D space is illustrated in Figure 3. The contrastive learning model was able to separate the post-COVID-19 subjects from the healthy controls and group them together by their similarity. Figure 4 demonstrated two representative subjects for each cluster that were closest to the respective cluster’s geometric centroids.

**FIGURE 5:**
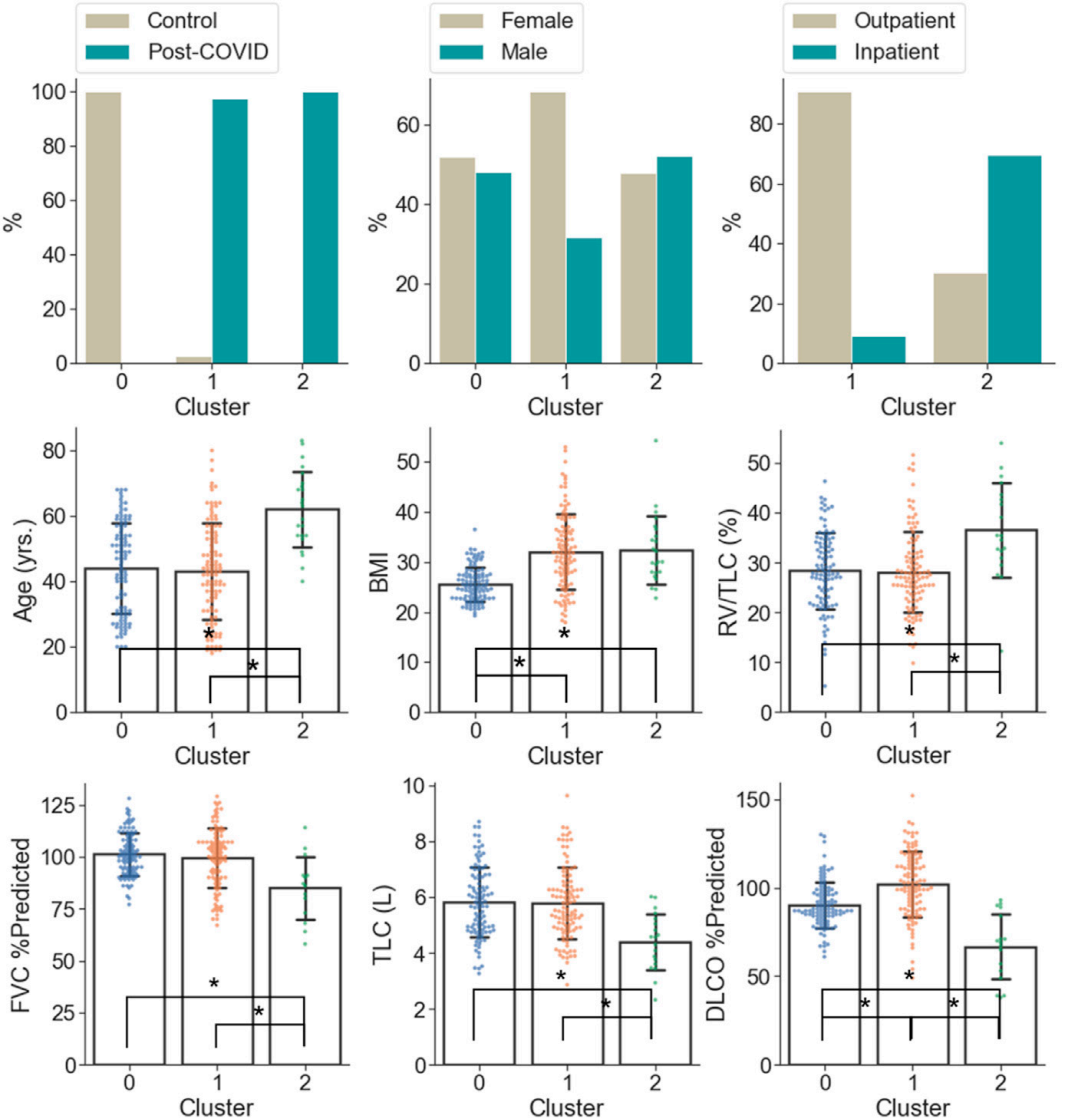
Demographic and PFT data which were significantly different (α = 0.05) between the clusters. A “*” denotes significance between two clusters and the range of error bar is the mean ± the standard deviation.

C0 consisted of the healthy controls only (0% post-COVID-19 subjects), while C1 and C2 were majorly composed of the post-COVID-19 subjects (97.50% and 100.00% post-COVID-19 subjects, respectively). C1 was female dominant (67.50% females) while C0 and C2 had relatively balanced numbers in females and males. The encounter types for COVID-19 diagnosis for C1 and C2 were mostly outpatient (90.37%) and inpatient (67.39%), respectively. There were significant differences between clusters in terms of age and BMI. C2 (62.04 yrs.) had greater age than C0 (44.15 yrs., *p* < 0.001) and C1 (43.59 yrs., *p* < 0.001). C1 (32.99, *p* < 0.001) and C2 (32.37, *p* < 0.001) had higher BMI than C0 (25.57) (Figure 5 and Supplementary Table S2). There was no significant difference between C1 and C2 in terms of the time interval between the diagnosis of COVID-19 and the first visit to the post-COVID-19 clinic (C1: 118.88 days, C2: 93.05 days, *p* = 0.199).

In terms of PFT results (Figure 5 and Supplementary Table S2), C2 was characterized by lower FVC %predicted (84.93%), DLCO %predicted (66.76%), TLC (4.40 L), and higher RV/TLC (36.48%) than C0 (FVC %predicted: 101.14%; *p* = 0.005, DLCO %predicted: 90.32%; *p* < 0.001, TLC: 5.87 L; *p* < 0.001) and C1 (FVC %predicted: 99.41%; *p* = 0.012, DLCO %predicted: 101.95%; *p* < 0.001, TLC: 5.77 L; *p* < 0.001). In addition, C1 was marked by the greatest DLCO %predicted (101.95%; *p* < 0.001 and *p* < 0.001 for C1 vs. C0 and C1 vs. C2, respectively) among the clusters.

**FIGURE 6:**
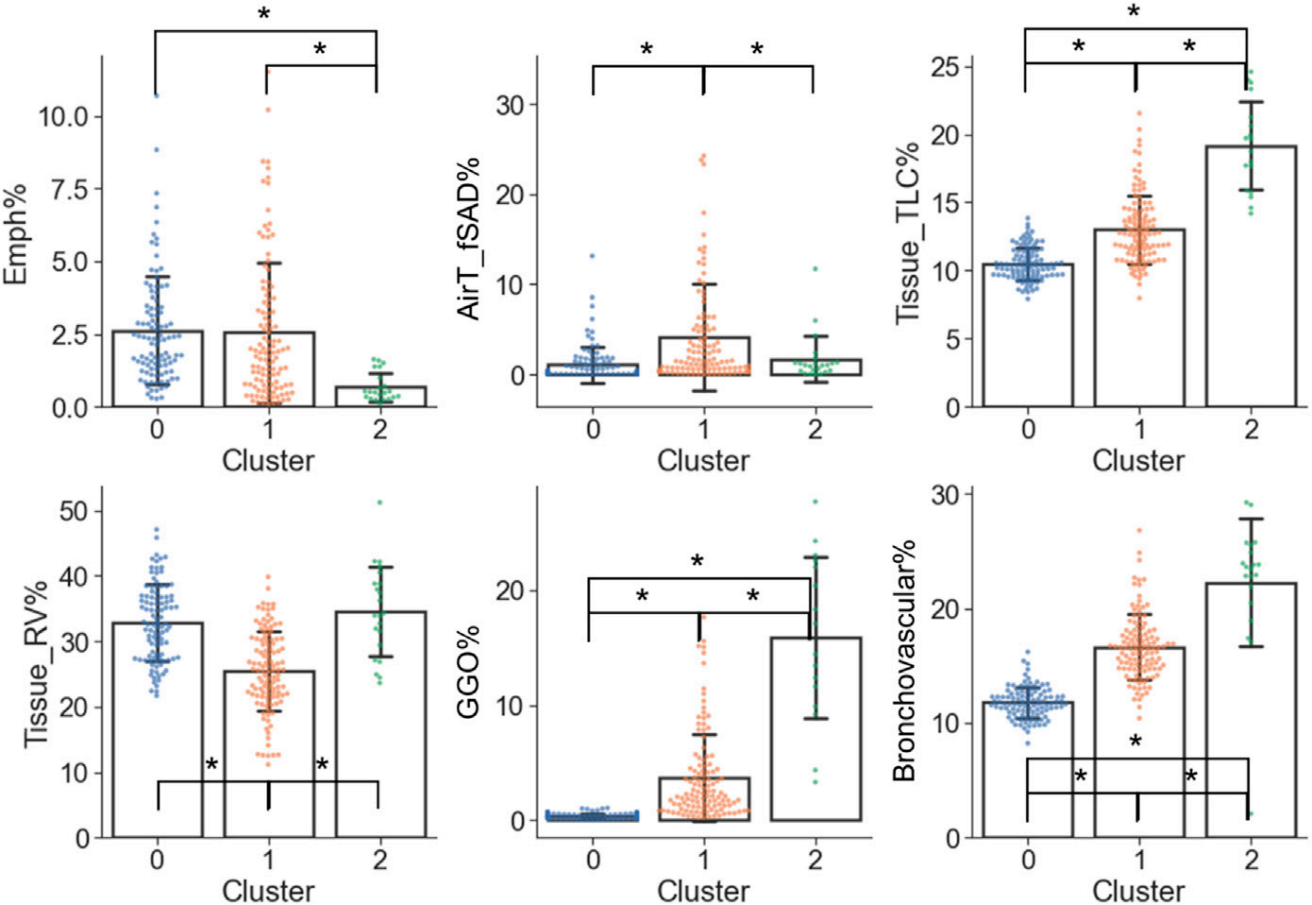
Imaging variables which were significantly different (α = 0.05) between the clusters. A “*” denotes significance between two clusters and the range of error bar is the mean ± the standard deviation.

From the perspective of imaging characteristics (Figure 6 and Supplementary Table S2), C2 had the greatest Tissue_TLC% (19.15%, *p* < 0.001 and *p* < 0.001 for C2 vs. C0 and C2 vs. C1, respectively), GGO% (15.85%, *p* < 0.001 and *p* < 0.001 for C2 vs. C0 and C2 vs. C1, respectively), Bronchovascular% (22.25%, *p* < 0.001 and *p* < 0.001 for C2 vs. C0 and C2 vs. C1, respectively), and the least Emph% (0.68%, *p* < 0.001 and *p* < 0.001 for C2 vs. C0 and C2 vs. C1, respectively). C1 had the highest AirT_fSAD% (4.10%, *p* < 0.001 and *p* = 0.008 for C1 vs. C0 and C1 vs. C2, respectively), and the lowest Tissue_RV% (25.55%, *p* < 0.001 and *p* < 0.001 for C1 vs. C0 and C1 vs. C2, respectively). Moreover, C1 had larger Tissue_TLC% (13.02%, *p* < 0.001), GGO% (3.68%, *p* < 0.001), and Bronchovascular% (16.64%, *p* < 0.001) than C0 (Tissue_TLC%: 10.50%, GGO%: 0.39%, Bronchovascular%: 11.79%). There was no significant difference between C0 and C2 in terms of AirT_fSAD% (C0: 1.08%, C2: 1.68%, *p* = 0.55), and Tissue_RV% (C0: 32.84%, C2: 34.63%, *p* = 0.51).

## Discussion

In this study, we proposed a volume-independent contrastive learning model to differentiate the images of the post-COVID-19 subjects from those of the healthy controls with an accuracy of 0.90 and an AUC of 0.98 on test data, and extract latent representations of the images for discovering subgroups of the post-COVID-19 subjects. Beside the inspiration images, expiration images were used to augment the training images so that the model can be generalized to different lung volumes. This was achieved through a lung volume transform in the training process to maximize the similarity between the inspiration and expiration images. The inclusion of the lung volume transform in the training process ensured the model to capture the features from the inspiration and expiration images of the same subjects. Without use of the lung volume transform, the model became volume dependent. The volume-independence model input feature is essential when precise volume control during scanning cannot be guaranteed.

Three clusters were identified by the latent representations extracted by the contrastive learning model. The first cluster C0 was regarded as the healthy control cluster since it was composed of healthy controls (0% post-COVID-19 subjects), while C1 and C2 clusters were treated as the post-COVID-19 subtypes since they consisted of mostly post-COVID-19 subjects with distinct clinical and imaging features (97.50% and 100.00% post-COVID-19 subjects for C1 and C2, respectively). The characteristics of C1 and C2 were summarized in Table 2.

**TABLE 2:** Summary of the cluster characteristics. A “+” and a ‘−’ denote that the Post-COVID-19 clusters were significantly greater and less than the control group in terms of the given variables.

	C1	C2
**Age**		+
**BMI**	+	+
**FVC %Predicted**		−
**DLCO %Predicted**	+	−
**RV/TLC**		+
**TLC**		−
**AirT_fSAD%**	+	
**Tissue_TLC %**	+	++
**Tissue_RV %**	−	+
**GGO %**	+	++
**Bronchovascular %**	+	++

C1 was dominated by obese female subjects with normal lung functions other than a higher lung diffusing capacity, and most of the C1 subjects were not hospitalized. C1 was characterized by increased lung tissue during inspiration, greater amount of GGO patterns, and thickening bronchovascular structure, which are known phenotypes associated with the inflammation caused by the infection of COVID-19. Another study has suggested that even in mild cases, return of increased GGO can still be observed after 90 days from the diagnosis of COVID-19 (Nagpal et al., 2021). Furthermore, increased air-trapping due to small airways disease (AirT_fSAD% ↑, Tissue_RV % **↓**) was found in C1 subjects. It has been shown that small airways disease, which is presented as air-trapping without emphysema, is a long-term sequela of COVID-19 (Cho et al., 2022; Jia et al., 2022). Small airways disease and emphysema are two common progressive phenotypes in patients with chronic obstructive pulmonary disease (COPD). The extent of AirT_fSAD% (or emphysema) in C1 subjects is comparable to (or lower than) that of the COPD C1 subjects in former smokers whose severity levels were predominately classified as at risk with GOLD stage 0. Normal DLCO is defined as 75%–140% of predicted (Ponce and Sharma, 2021), so the DLCO % predicted of post-COVID-19 subjects is within the normal range for healthy subjects. The slightly elevated DLCO %predicted in post-COVID-19 subjects was contributed by C1 subjects (101.95%). On the other hand, C2 subjects has decreased and abnormal DLCO %predicted (66.76%). It has been found in a retrospective study that the elevated lung diffusing capacity may related to a clinical diagnosis of obesity and asthma (Saydain et al., 2004). C1 subjects may share similar characteristics with obesity and asthma subjects. It requires more investigation in the future to better understand the elevated DLCO %predicted in C1 subjects.

On the other hand, C2 was dominated by older subjects with impaired lung functions and a more rigid lung (RV/TLC↑, TLC↓), and most of them were hospitalized due to COVID-19. The fact that C2 demonstrated the greatest lung tissue content observed from both expiration and inspiration images and the greatest amount of GGO patterns may show the signs of interstitial fibrotic-like patterns (Supplementary Figure S2). Moreover, increased bronchovascular thickening (Bronchovascular % ↑) may indicate early bronchiectasis. The hot spots shown in the activation maps demonstrated the features of thickened bronchovascular structures which were observed in C2 (Supplementary Figure S2). It is well established that age and obesity are two of the major risk factors of severe COVID-19 (Zheng et al., 2020; Wolff et al., 2021). Furthermore, it has been reported that severity of COVID-19 is a risk factor of fibrotic-like patterns in post-COVID subjects (McGroder et al., 2021). Thus, C2 may represent the subjects who suffered badly from COVID-19.

This study had several limitations. First, the sample size is relatively small and the cohort came from a single center. There may exist more subgroups of post-COVID-19 subjects due to comorbidities, which would require a larger sample size to be identified. To enhance the generalizability of the clusters, active learning models which learn the multimodal and cross-population data over time would be beneficial (Santosh, 2020; Santosh and Ghosh, 2021). Second, there was no reading for the clusters from radiologists, which may provide more insight to the interpretation of the clusters. In addition, this is a cross-sectional study. The progression of these subtypes requires further investigation.

In this study, we demonstrated that a volume-independent contrastive learning model can differentiate the CT images of post-COVID-19 subjects from those of healthy controls, and it can also extract distinct latent representations from the images for clustering post-COVID-19 subjects. We identified two clinically meaningful subtypes among the post-COVID-19 study cohort. Clusters C1 and C2 are characterized by subjects with air-trapping caused by small airways disease and subjects with airway-associated interstitial fibrotic-like patterns, respectively. It has been reported that contrastive models are able to achieve better performance on unseen data than traditional deep learning models (Chen et al., 2020). With the design of 2D image input, we expect that this pre-trained model can be used for transfer learning on chest X-ray images, which are more accessible at regular clinics, to detect and classify patients with post-COVID-19. Furthermore, with increasing cohort size the model has potential to detect the features caused by different COVID-19 variants. Finally, the knowledge of clinical and imaging features of post-COVID-19 subtypes and the ability of subject classification by the model may facilitate the management and treatment of long COVID.

## Data Availability

The data analyzed in this study is subject to the following restriction: the raw CT scans may contain identifiable human information. As such, any request for data access shall be sent to Professor AC, Department of Internal Medicine, University of Iowa (alejandro-comellas@uiowa.edu).
