# Supplementary material for: Contrastive learning and subtyping of post-COVID-19 lung computed tomography images
Source: Front Physiol. 2022 Oct 11;13:999263. doi: 10.3389/fphys.2022.999263 (PMC9593072; doi:10.3389/fphys.2022.999263)
Supplement: Supplementary file 1 [file DataSheet1.docx]

Supplementary Material

# Supplementary Figures
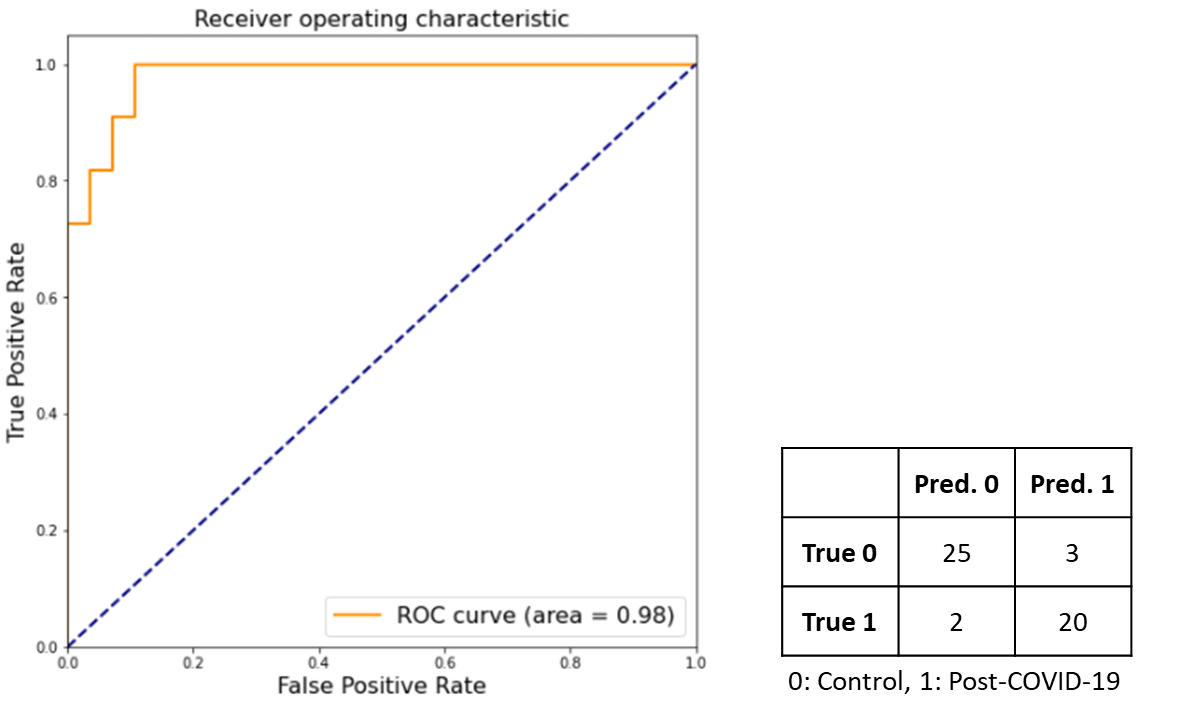
Supplementary Figure 1. The receiver operating characteristic curve of our contrastive learning model.


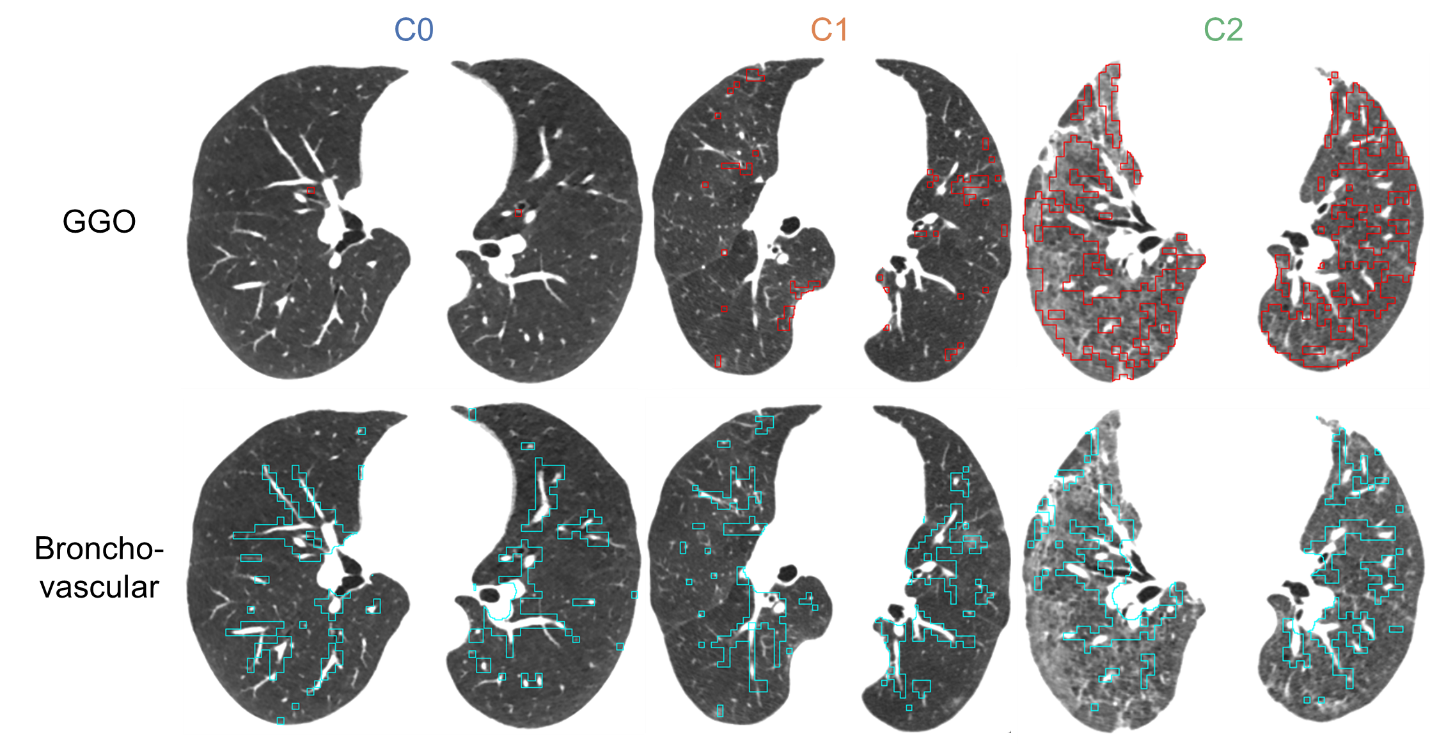


**Supplementary Figure 2.** Cross-sectional CT images of the representative subjects of the clusters plotted with a level of -700 HU and a window of 800 HU. The GGO and bronchovascular patterns were shown within the red and cyan bounding boxes, respectively.

# Supplementary Tables

# Supplementary Table 1. Numbers of missing data for the variables analyzed in this study.

|  | **Number of Missing data** | **Note** |
| --- | --- | --- |
| **Encounter Type for Covid Diagnosis** | 5 | For Post-COVID-19 Subjects only |
| **Interval** | 2 | For Post-COVID-19 Subjects only |
| **Gender** | 0 |  |
| **Age (yrs.)** | 0 |  |
| **BMI** | 2 |  |
| **FVC %Predicted** | 14 |  |
| **FEV1 %Predicted** | 13 |  |
| **RV/TLC (%)** | 13 |  |
| **TLC (L)** | 12 |  |
| **RV (L)** | 12 |  |
| **DLCO %Predicted** | 11 |  |
| **Emph%** | 0 |  |
| **AirT%** | 0 |  |
| **Tissue_TLC%** | 0 |  |
| **Tissue_RV%** | 0 |  |
| **Ground Glass** | 0 |  |
| **fSAD%** | 0 |  |
| **Bronchovascular%** | 0 |  |

**Supplementary Table 2.** A table that summarizes the data in Figure 5 and Figure 6.

|  | **Controls** | **Cluster 1** | **Cluster 2** |
| --- | --- | --- | --- |
| **Emph%** | 2.62 (1.86) | 2.56 (2.41) | 0.68 (0.49) |
| **fSAD%** | 1.08 (1.97) | 4.10 (5.94) | 1.68 (2.59) |
| **Tissue_TLC%** | 10.50 (1.22) | 13.02 (2.52) | 19.15 (3.32) |
| **Tissue_RV%** | 32.84 (5.86) | 25.55 (6.11) | 34.63 (6.94) |
| **GGO%** | 0.39 (0.23) | 3.68 (3.80) | 15.85 (7.20) |
| **Bronchovascular%** | 11.78 (1.37) | 16.64 (2.84) | 22.25 (5.72) |
| **Sex (Female %)** | 51.96 | 68.38 | 47.83 |
| **Age (yrs)** | 44.15 (13.93) | 43.06 (14.76) | 62.04 (11.85) |
| **BMI** | 25.57 (3.48) | 32.05 (7.65) | 32.37 (6.95) |
| **FEV1% pred** | 99.54 (10.76) | 98.69 (14.27) | 89.14 (15.27) |
| **FVC% pred** | 101.14 (10.40) | 99.41 (14.30) | 84.93 (15.68) |
| **FEV1/FVC x100** | 79.00 (5.86) | 81.31 (6.31) | 81.65 (4.55) |
| **DLCO% pred** | 90.31 (12.99) | 101.95 (18.56) | 66.76 (18.76) |
| **RV/TLC** | 28.31 (7.79) | 28.05 (8.14) | 36.48 (9.65) |
| **TLC (L)** | 5.83 (1.26) | 5.77 (1.28) | 4.40 (1.02) |

# Supplementary Result

Two logistic regression models were built using GGO% and Bronchovascular%, which were found to be significant different between clusters, as predictors for differentiating post-COVID-19 from healthy subjects on test dataset. They were able to achieve accuracies of 92% (AUC =0.95) and 88% (AUC =0.95) using GGO% and Bronchovascular% as predictors, respectively. The performance of the logistic regression models is comparable to the proposed contrastive learning model (ACC=90%, AUC=0.98). However, the proposed model is able to extract features for clustering as well as prediction of pPost-COVID-19 subjects without subjective feature selection.
